# d-Lactic acid production from dry biomass of *Hydrodictyon reticulatum* by simultaneous saccharification and co-fermentation using *Lactobacillus coryniformis* subsp. *torquens*

**DOI:** 10.1007/s10529-012-1023-3

**Published:** 2012-08-30

**Authors:** Cuong Mai Nguyen, Jin-Seog Kim, Jae Kwang Song, Gyung Ja Choi, Yong Ho Choi, Kyoung Soo Jang, Jin-Cheol Kim

**Affiliations:** 1 University of Science and Technology, Research Center for Biobased Chemistry, Korea Research Institute of Chemical Technology, Daejeon, 305-600 Republic of Korea; 2 Research Center for Biobased Chemistry, Korea Research Institute of Chemical Technology, Daejeon, 305-600 Republic of Korea

**Keywords:** d-Lactic acid, *Hydrodictyon reticulatum*, *Lactobacillus coryniformis* sub. *torquens*, Simultaneous saccharification and co-fermentation

## Abstract

d-Lactic acid production from dry biomass of the microalga, *Hydrodictyon reticulatum,* was carried out in a 5-l jar fermentor (initial pH 6, 34 °C using CaCO_3_ as a neutralizing agent) through simultaneous saccharification and co-fermentation using the *Lactobacillus coryniformis* subsp. *torquens.* After 36 h, 36.6 g lactic acid/l was produced from 80 g *H. reticulatum*/l in the medium containing 3 g yeast extract/l and 3 g peptone/l in the absence of mineral salts. The maximum productivity, average productivity and yield were 2.38 g/l h, 1.02 g/l h and 45.8 %, respectively. The optical purity of d-Lactic acid ranged from 95.8–99.6 %. *H. reticulatum* is thus a promising biomass material for the production of d-Lactic acid.

## Introduction

Lactic acid (LA) is widely used in the food, pharmaceutical, leather, textile and cosmetic industries and has been regarded as one of the top 30 potential building-block chemicals from biomass (Coelho et al. 2011; Werpy and Petersen 2004). In particular, LA has gained considerable attention because of its use as a major raw material for the manufacture of polylactic acid (PLA), the biodegradable synthetic polyester. The current worldwide demand for lactic acid is estimated roughly to be 130,000–150,000 tons per year (Wee et al. 2006). The chemical synthesis of LA is achieved industrially using petrochemicals as common raw materials, which produced a racemic mixture of l- or d-LA, whereas microbial fermentation has advantages in both the utilization of renewable resources and the production of optically pure isomers. Production of optically pure isomers is important for industrial use because a careful blending of both isomers provides an effective way of controlling the physical properties and the biodegradability of PLA. A stereocomplex of PLA produced by blending poly(l-LA) (PLLA) and poly(d-LA) (PDLA) had a much higher melting temperature (approximately 230 °C) than PLLA (Fukushima et al. 2007; Tsuji and Fukui 2003). Therefore, the microbial production of pure d-LA has now become increasingly more important, in addition to l-LA, which has been the focus of many studies to date.

Cheap raw materials, such as starchy and cellulosic materials and molasses, have been used for LA production. Among these, starchy and cellulosic materials are currently receiving a great deal of attention, because they are cheap, abundant, and renewable (John et al. 2009; Joshi et al. 2010; Wee et al. 2006). A promising development in the conversion of lignocellulosic biomass to renewable fuels and chemicals are the green microalgae. Microalgae grow almost anywhere and have an extremely short harvesting cycle of approximately 1–10 days (Schenk et al. 2008). The fresh water microalgae *Hydrodictyon reticulatum* (HR), containing mainly glucose and mannose, has many advantages as a feedstock with a high polysaccharide content over other biomass sources such as sugar cane, rice, corn, cassava, etc. because of its ability to be produced quickly and cheaply with productivity 20 ton/ha. Therefore, HR can be used as a raw material for LA production (Nguyen et al. 2012). This paper describes the production of d-LA from HR through simultaneous saccharification and co-fermentation (SSCF) by *Lactobacillus coryniformis* subsp. *torquens* which is a homofermentative d-LA producer (Yanez et al. 2003).

## Materials and methods

### Raw material and enzymes

The alga (*Hydrodictyon reticulatum:* HR) was washed in tap water, air-dried (water content: 6 %) and stored in plastic bags at 4 °C in a dry, dark place until use. HR contained 1.4 % lipid, 10 % protein and 62.9 % carbohydrate including 15.8 % starch (% w/w).

Celluclast Conc BG (CC100133), with an activity of 385 FPU/g, was from Novozymes in solid form and 1 g powder was dissolved in 5 ml of 1 M citrate buffer (pH 4.8) (64 FPU/ml; E1). Cellobiase (C6105: E2) from *Aspergillus niger* was from Sigma-Aldrich; its β-glucosidase activity was 311 U/ml. α-Amylase Fungamyl 800 L (1,4-α-d-glucan glucanohydrolase, EC 3.2.1.1: E3) from *A. oryzae* and amyloglucosidase (A7095: E4) from *A. niger* were from Sigma-Aldrich in the liquid form their activities were 800 FAU/g and 300 U/ml, respectively. S1, S2, S3, S4, S5, and S6 were mixtures containing the four enzymes in ratios of 2/2/2/2, 2/2/0/2, 2/0/0/2, 2/1/0/1, 2/0/0/0, and 1/1/0/1 (by vol.) of E1/E2/E3/E4, respectively; the numbers indicate the amounts of the enzymes as percentages of the dried biomass material (v/w). The activities of the enzymes reported here are those claimed in the description found in the supplier’s product sheets.

### Microorganism and culture medium

*Lactobacillus coryniformis* sub. *torquens* ATCC 25600 was grown on MRS medium at 34 °C with agitation at 220 rpm for 20 h. The effects of the enzyme mixture, nitrogen source and HR concentration on LA production from HR were analyzed in the medium, which contained 0.05 g MnSO_4_, 2 g K_2_HPO_4_, 5 g CH_3_COONa, 0.2 g MgSO_4_, 2 g triammonium citrate, 1 g Tween 80 and 25 g CaCO_3_ per liter. The medium for the jar fermentor contained 3 g yeast extract (YE)/l, 3 g peptone/l and 25 g CaCO_3_/l. The initial pH was adjusted to 6 using 2 M H_2_SO_4_.

### Optimum conditions in 50 ml Erlenmeyer flasks

To determine the optimum enzyme compositions and the HR concentrations for the production of d-LA through SSCF using strain ATCC 25600, six different enzyme mixtures, S1–S6, were tested at 80 g HR/l. The effects of the HR concentration were carried out at 60, 80, 100, and 120 g HR/l using the S4 mixture. The effect of the YE on LA production from the HR material was performed at 2, 3, 5, and 7 g YE/l supplemented with 0, 3, and 5 g peptone/l. The effect of the mineral salts was evaluated at 3 g YE/l and 3 g peptone/l without any mineral salt addition. The pre-culture and enzyme mixture were inoculated into the fermentation medium at 10 % (v/v) and 4 % (v/w) levels, respectively, and then incubated under anaerobic conditions in a vinyl anaerobic chamber at 34 °C with agitation at 220 rpm using 50 ml Erlenmeyer flasks containing 15 ml medium.

### Lactic acid production from HR using a jar fermentor

SSCF was carried out in a 5-l jar fermentor with a working volume of 2 l under conditions selected in 50 ml Erlenmeyer flasks. After autoclaving at 121 °C for 21 min, 1.8 l of the medium containing 160 g HR material, 6 g YE, 6 g peptone, and 50 g CaCO_3_ was used as the SSCF medium. The SSCF experiments were initiated by the addition of 6.4 ml of the S4 enzyme mixture and inoculation with 200 ml pre-culture of *L. coryniformis* sub. *torquens*. The temperature, agitation speed and dissolved O_2_ were maintained at 34 °C, 600 rpm, and ≤0.5, respectively.

### Analytical methods

Cell-forming unit values were determined by the dilution spread plate method on MRS agar medium (diluted 10^−6^ to 10^−8^). To determine the LA and glucose levels, the samples were heated at 100 °C for 10 min, diluted to 0.1 % and then centrifuged at ~6,000 × *g* and 37 °C for 20 min. Calcium lactate was hydrolyzed to LA at pH 2 using 5 M H_2_SO_4_, and then the samples were diluted to 0.01–0.3 g/l (l- or d-LA) and 0.1–0.5 g/l (glucose) for analysis. l-LA and d-glucose concentrations were measured using an YSI 2700 Select Biochemistry Analyzer. d-LA was quantified using an enzymatic kit (Megazyme K-DATE 06/08, Megazyme International Ireland Ltd.). Levels of reducing sugars (RS) in the culture broths, RS conversion values and yield were estimated and calculated following the method previously described (Nguyen et al. 2012).

## Results and discussion

**Fig. 1:**
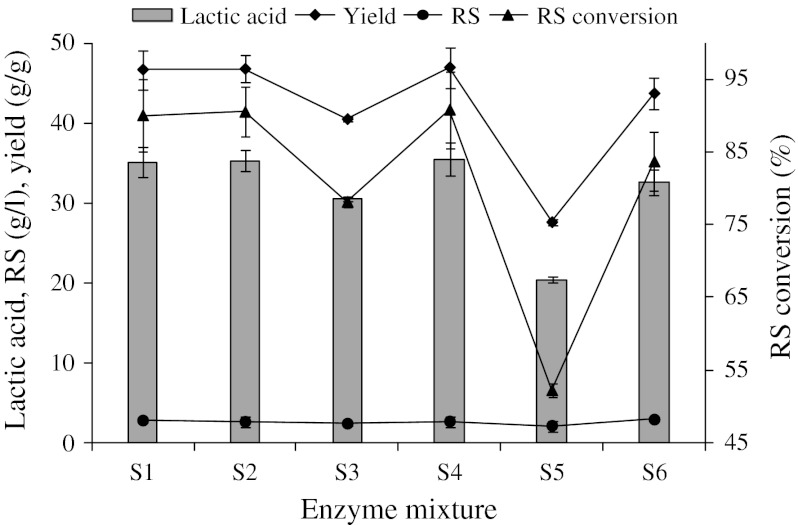
Effect of different enzyme mixtures on simultaneous saccharification and co-fermentation of lactic acid from *Hydrodictyon reticulatum* by *Lactobacillus coryniformis* subsp. *torquens* ATCC 25600. The experiment was performed at 34 °C with agitation at 220 rpm, for 48 h in the medium containing 80 g HR/l, 10 g YE/l, 5 g peptone/l 0.05 g MnSO_4_/l, 2 g K_2_HPO_4_/l, 5 g sodium acetate, 0.2 g MgSO_4_/l, 2 g triammonium citrate/l, and 1 g Tween 80/l. *RS* reducing sugar

Figure 1 shows the effects of enzyme concentration and enzyme mixtures on the SSCF process. The enzyme effect was first investigated at fixed conditions (initial pH 6, 34 °C, 80 g HR/l, 220 rpm and 48 h) and its effect was significant. LA increased from 20.4 g/l (corresponding to a 25.5 % yield) for the sample treated with S5 enzyme mixture to 35.4 g/l (corresponding to a 44.3 % yield), for the sample treated with the S4 enzyme mixture. There is no doubt that the increase in enzyme concentration and enzyme mixture would normally enhance hydrolysis, resulting in increased LA yields. LA concentrations and yields from the S1, S2, and S4 mixtures were higher than those from the S3, S5, and S6. No significant differences were observed among the S1, S2 and S4 mixtures.

Enzyme concentration is one of the factors contributing to production costs; therefore, the S4 mixture is the most preferred choice for SSCF. This study indicates that d-LA production from HR using *L. coryniformis* sub. *torquens* ATCC 25600 through the SSCF does not require α-amylase like in the case of l-LA production from HR using *L. paracasei* LA104 (Nguyen et al. 2012). Compared to the production of l-LA from HR using *L. paracasei* LA104 through the SSCF process, the amounts of cellobiase and amyloglucosidase decreased by 50 %.

As shown in Table 1, the maximum yield was 46.0 % (corresponding to a 94.3 % RS conversion) obtained when SSCF was conducted at 60 g HR/l. The final RS conversions were 94.1, 88.2 and 87.5 % at 80, 100 and 120 g HR/l, respectively. The results indicated that the efficiency of LA production using the S4 mixture was relevant to the concentration of the material. The low RS conversions and low yields at high HR concentrations may be ascribed to the high viscosity, uneven slurry distribution and end-product inhibition of degradation activity of enzymes, but not to the toxicity of LA to the cell because RS in the culture broth were not significantly different for all of HR concentrations. According to Iyer and Lee (1999), the extent of the inhibition is such that the enzymatic digestibility at 72 h decreased from 79 to 56 % as the LA concentration increased from 0 to 90 g/l. Moreover, the concentration of fermentable sugars in the medium is important to the subsequent LA concentration. Therefore, to optimize the enzymatic hydrolysis of HR, both the RS conversion efficiency and LA concentration in the culture broth should be considered. Therefore, in all subsequent SSCF experiments, we used 80 g HR/l.

**Table 1:** Effect of *Hydrodictyon reticulatum* (HR) concentration on d-Lactic acid production

HR concentration (g/l)	Lactic acid (g/l)	Reducing sugar (HR) (g/l)	Yield (%)	Reducing sugar conversion (%)	Optical purity (%)
60	27.6 ± 0.46	2.68 ± 0.15	46 ± 0.76	94.3 ± 0.76	99.4 ± 0.02
80	36.7 ± 0.87	2.95 ± 0.45	45.9 ± 1.09	94.1 ± 1.09	99.5 ± 0.03
100	43 ± 1.62	3.7 ± 0.01	43 ± 1.62	88.2 ± 1.62	99.4 ± 0
120	51.2 ± 2.87	4.08 ± 0	42.7 ± 2.39	87.5 ± 2.39	99.5 ± 0.05

The cultures were incubated at 34 °C, with agitation at 220 rpm for 48 h using the S4 enzyme mixture. The values are given as the average of four independent experiments

Using medium containing 80 g HR/l, LA increased with the addition of YE and peptone, and 36.7 g LA/l (corresponding to 45.9 % yield) was obtained when 3 g YE/l was supplemented with 3 g peptone/l. Further addition of YE and peptone did not significantly improve LA production. YE is most commonly used as a rich nutrient source of vitamin B and amino acids in fermentations, and can enhance LA production rates by LA bacteria; however, high prices hinder its large scales use. In an economic analysis, the largest contributor for LA production cost was found to be YE, which generally accounted for approximately 38 % of the total production medium cost (Teleyadi and Cheryan 1995). In this study, low amounts of YE and peptone were required for the production of d-LA from HR.

In addition, LA concentrations in SSCF, which were estimated in the media in the presence or absence of MnSO_4_, were almost the same as that observed in the presence of various mineral salts, including MnSO_4_ (Table 2). Compared with l-LA production from HR using *L. paracasei* (Nguyen et al. 2012), the fermentation process using *L. coryniformis* subsp. *torquens* did not require any additional mineral salts. Therefore, the optimum culture condition for the production of d-LA by *L. coryniformis* subsp. *torquens* ATCC 25600 was determined to be: 80 g HR/l, 3 g YE/l, 3 g peptone/l, and the presence of the S4 enzyme mixture.

**Table 2:** Effects of different nitrogen sources and mineral salts on d-Lactic acid production after 36 h

Nitrogen source and mineral salts	Lactic acid (g/l)	Reducing sugar remaining (g/l)	Reducing sugar conversion (%)^d^	Yield (%)^e^	Optical purity (%)^f^
2 g YE^a^	26.8 ± 1.19	11.5 ± 0.88	68.5 ± 1.53	33.4 ± 1.49	99.6 ± 0.02
2 g YE and 3 g peptone^a^	32.2 ± 0.12	6.53 ± 0.70	82.6 ± 0.15	40.3 ± 0.15	99.7 ± 0
3 g YE^a^	34.4 ± 0.57	2.96 ± 0.16	88.1 ± 0.74	42.9 ± 0.72	99.6 ± 0.21
3 g YE and 3 g peptone^a^	36.7 ± 0.34	2.93 ± 0.33	94.2 ± 0.44	45.9 ± 0.43	99.7 ± 0.01
5 g YE^a^	36.5 ± 0.57	2.89 ± 0.49	93.7 ± 0.73	45.7 ± 0.71	99.7 ± 0.04
5 g YE and 3 g peptone^a^	36 ± 0.01	3.15 ± 0.02	92.2 ± 0.01	45.0 ± 0.01	99.7 ± 0.02
5 g YE and 5 g peptone^a^	36.2 ± 0.31	3.1 ± 0.12	92.9 ± 0.39	45.3 ± 0.38	99.6 ± 0.02
7 g YE and 5 g peptone^a^	36.8 ± 0.42	3.0 ± 0.21	94.2 ± 0.54	45.9 ± 0.53	99.6 ± 0.02
3 g YE and 3 g peptone^b^	37.0 ± 1.21	2.85 ± 0.66	94.8 ± 1.55	46.2 ± 1.51	99.6 ± 0.03
3 g YE and 3 g peptone^c^	36.7 ± 0.84	2.96 ± 0.5	94.2 ± 1.08	45.9 ± 1.05	99.6 ± 0.04

^a^The mineral salts consisted of 0.05 g MnSO_4_, 2 g K_2_HPO_4_, 5 g sodium acetate, 0.2 g MgSO_4_, 2 g triammonium citrate and 1 g Tween 80

^b^The medium contained yeast extract (YE) as a nitrogen source and MnSO_4_ as a mineral salt

^c^The medium contained only YE as a nitrogen source

^d^Lactic acid (g/l) × 100/reducing sugar in the *Hydrodictyon reticulatum* material (g/l) ± SD

^e^Lactic acid (g/l)/material (g/l) ± SD

^f^ d-Lactic acid (g/l) × 100/(l-Lactic acid [g/l] + d-Lactic acid [g/l]) ± SD

**Fig. 2:**
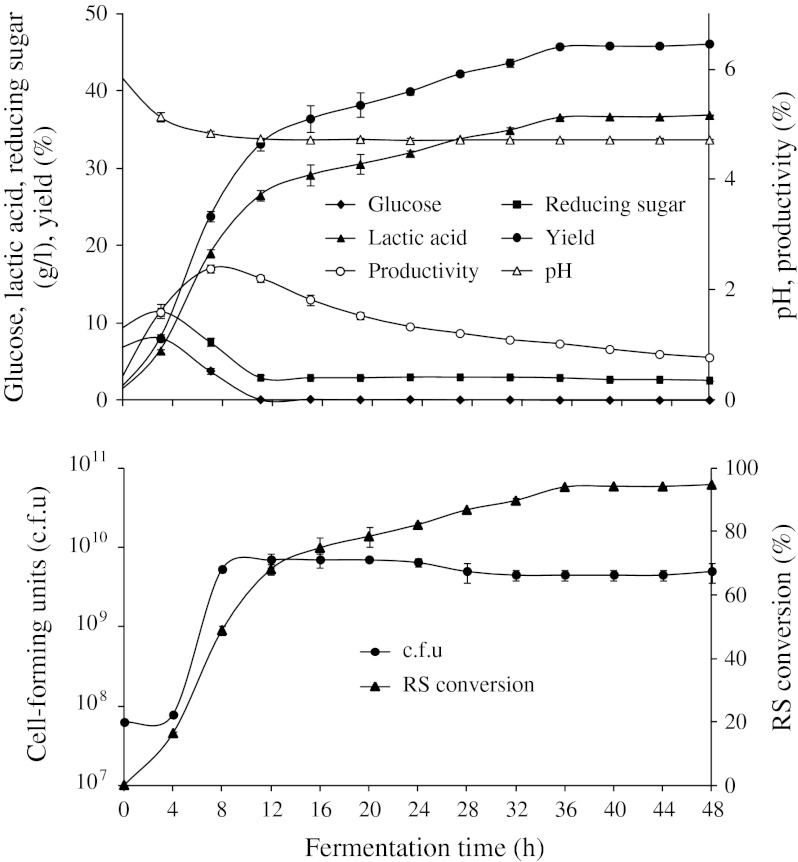
Time course of glucose, lactic acid, reducing sugar (*RS*) concentrations, cell-forming units (c.f.u), pH, productivity, reducing sugar conversion, and yield in SSCF experiments using *Lactobacillus coryniformis* subsp. *torquens* ATCC 25600 performed in medium containing 80 g *Hydrodictyon reticulatum*/l, 3 g YE/l and 3 g peptone/l in the absence of any mineral salts using a 5-l jar fermentor

To confirm the optimum fermentation condition, an experiment was performed in a jar fermentor. The average productivity, maximum productivity and yield were 1.02 g/l h, 2.38 g/l h, and 45.8 %, respectively (Fig. 2). The RS remaining was 2.91 g/l after 12 h, which did not decrease with the extension of the fermentation time. Most of the RS were converted to LA after 36 h. During the fermentation, the optical purity increased from 95.8 to 99.6 % and was steadily maintained after 16 h (data not shown). Few reports on d-LA production from cellulosic biomass through SSCF have been reported. Relatively low LA concentrations or low productivities were obtained when cellulose (Yanez et al. 2003), waste cardboard (Yanez et al. 2005), and defatted rice bran (Tanaka et al. 2006) were used for d-LA production through SSCF. Singhvi et al. (2010) reported that a yield of 0.73 g/g with a high productivity (1.52 g/l h) was obtained during LA production by SSCF using the *L. lactis* mutant RM2-24, however, in this case, the substrate was *α*-cellulose. Except d-LA production from α-cellulose using an *L. lactis* mutant (Singhvi et al. 2010), the productivities of d-LA from various cellulosic biomass materials through SSCF were lower than that of our study. In addition, we have previously reported l-LA production from HR using the *L. paracasei* strain LA104, which resulted in high productivity and yield values of 1.03 g/l h and 46 %, respectively. Therefore, *H. reticulatum* is a promising biomass material for d- or l-LA production.

## Conclusion

A higher productivity and yield of d-LA from *H. reticulatum* by *L. coryniformis* subsp. *torquens*, compared to other renewable substrates, has been shown. The fermentation process required small amounts of YE and peptone but no additional mineral salts. Thus, *H. reticulatum* could be a potential feedstock for large-scale production of d-LA through the SSCF by *L. coryniformis* subsp. *torquens* using a simple fermentation medium.
